# Primary congenital glaucoma localizes to chromosome 14q24.2-24.3 in two consanguineous Pakistani families

**Published:** 2008-09-05

**Authors:** Sabika Firasat, S. Amer Riazuddin, J. Fielding Hejtmancik, Sheikh Riazuddin

**Affiliations:** 1 National Centre of Excellence in Molecular Biology, University of the Punjab, Lahore Pakistan; 2 Ophthalmic Genetics and Visual Function Branch, National Eye Institute, National Institutes of Health, Bethesda, MD

## Abstract

**Purpose:**

Two consanguineous Pakistani families with autosomal recessive primary congenital glaucoma were recruited to identify the disease locus.

**Methods:**

Ophthalmic examinations including slit lamp biomicroscopy and applanation tonometry were employed to classify the phenotype. Blood samples were collected and genomic DNA was extracted. A genome wide scan was performed on both families with 382 polymorphic microsatellite markers. Two point LOD scores were calculated, and haplotypes were constructed to define the disease interval.

**Results:**

Clinical records and ophthalmic examinations suggest that affected individuals in families PKGL005 and PKGL025 have primary congenital glaucoma. Maximum two-point LOD scores of 5.88 with D14S61 at θ=0 and 6.19 with D14S43 at θ=0 were obtained for families PKGL005 and PKGL025, respectively. Haplotype analysis defined the disease locus as spanning a 6.56 cM (~4.2 Mb) genetic interval flanked by D14S289 proximally and D14S85 distally.

**Conclusions:**

Linkage analysis localizes autosomal recessive primary congenital glaucoma to chromosome 14q24.2–24.3 in consanguineous Pakistani families.

## Introduction

Glaucoma is the second leading cause of vision loss, and approximately 15% of blindness worldwide result from glaucoma [1]. It is a group of poorly understood neurodegenerative disorders that are usually associated with elevated intraocular pressure [2]. Glaucoma is clinically and genetically heterogeneous with several different forms, each with diverse causes and severities. Clinically, it is characterized by slow but progressive degeneration of retinal ganglion cells and their axons, leading to deterioration of the visual field and to optic nerve atrophy.

Although rare, primary congenital glaucoma (PCG) is the most common form of glaucoma in infants with an overall occurrence of 1 in 10,000 births [3]. It is prevalent in countries where consanguinity is common with incidence as high as 1 in 1,250 births in the Slovak population, 1 in 2,500 births in Saudi Arabia, and 1 in 3,300 births in the state of Andhra Pradesh in India [4,5]. PCG is an inherited ocular congenital anomaly of the trabecular meshwork and anterior chamber angle [6-9]. This leads to the obstruction of aqueous outflow and increased intraocular pressure (IOP) resulting in optic nerve damage leading to childhood blindness. The disease manifests in the neonatal or early infantile period with symptoms of photophobia, epiphora, signs of globe enlargement, edema, opacification of the cornea, and breaks in Descemet's membrane. The mode of inheritance is largely autosomal recessive with variable penetrance, but rare cases of pseudo dominance are also seen in families with multiple consanguinity [10-13]. To date, three genetic loci have been reported for autosomal recessive PCG, *GLC3A* (2p21; OMIM 231300), *GLC3B* (1p36; OMIM 600975), and *GLC3C* (14q24.3), with pathogenic mutations only reported in the human cytochrome P450 gene (*CYP1B1*; OMIM 601771) [14,15]. It is significant to note that *CYP1B1* mutations have also been reported in patients with early onset of primary open-angle glaucoma. Additionally, autosomal dominant forms of PCG have been reported, and *MYOC*, a gene associated with primary open-angle glaucoma, is reported to play a possible role in the pathogenesis [16,17].

The current study is aimed to explore the genetic basis of PCG in the Pakistani population. A genome wide linkage analysis was performed, which showed segregation of PCG in two consanguineous Pakistani families. Microsatellite markers on chromosome 14q24.2–24.3 cosegregated with the disease phenotype and defined the disease locus as spanning a 6.56 cM (~4.2 Mb) genetic interval flanked by D14S289 proximally and D14S85 distally.

## Methods

Thirteen consanguineous Pakistani families with PCG were recruited to participate in this study to understand the genetic aspects of glaucoma at the Centre of Excellence in Molecular Biology (Lahore, Pakistan). Institutional Review Board approval was obtained for this study from the Centre of Excellence in Molecular Biology (CEMB). The participating subjects gave informed consent consistent with the tenets of the Declaration of Helsinki. Both families described in this study are from the Punjab province of Pakistan.

A detailed medical history was obtained by interviewing family members. All of the ophthalmic examinations including slit lamp biomicroscopy and applanation tonometry were completed at the Layton Rahmatullah Benevolent Trust (LRBT) hospital (Lahore, Pakistan). Diagnosis of PCG was based on established criteria that include measurement of IOP, measurement of corneal diameters, and observation of optic nerve head where possible as well as symptoms of corneal edema including photophobia, buphthalmos, cloudy cornea, and excessive tearing. Patients with elevated IOP associated with other systemic or ocular abnormalities were excluded. Blood samples were collected from affected and unaffected family members. DNA was extracted by a non-organic method as described by Grimberg et al. [18].

### Genotype analysis

A genome wide scan was performed with 382 highly polymorphic fluorescent markers from the ABI PRISM Linkage Mapping Set MD-10 (Applied Biosystems, Foster City, CA) having an average spacing of 10 cM. Multiplex polymerase chain reactions (PCRs) were performed in a 5 μl mixture containing 40 ng genomic DNA, various combinations of 10 μM dye labeled primer pairs, 0.5 μl 10X GeneAmp PCR Buffer II, 0.5 μl 10mM Gene Amp dNTP mix, 2.5 mM MgCl_2_, and 0.2 U of Taq DNA polymerase (AmpliTaq Gold Enzyme; Applied Biosystems). Amplification was performed in a GeneAmp PCR System 9700 (Applied Biosystems). Initial denaturation was performed for 5 min at 95 °C followed by 10 cycles for 15 s at 94 °C, for 15 s at 55 °C, and for 30 s at 72 °C, and then 20 cycles for 15 s at 89 °C, for 15 s at 55 °C, and for 30 s at 72 °C. The final extension was performed for 10 min at 72 °C followed by a final hold at 4 °C. PCR products from each DNA sample were pooled and mixed with a loading cocktail containing HD-400 size standards (PE Applied Biosystems). The resulting PCR products were separated in an ABI 3100 DNA Analyzer and analyzed by using the GeneMapper software package (Applied Biosystems).

### Linkage analysis

Two point linkage analysis were performed using the FASTLINK version of MLINK from the LINKAGE Program Package (provided in the public domain by the Human Genome Mapping Project Resources Centre, Cambridge, UK) [19,20]. Maximum LOD scores were calculated using ILINK. Autosomal recessive PCG was analyzed as a fully penetrant trait with an affected allele frequency of 0.001. The marker order and distances between the markers were obtained from the Marshfield database. For the initial genome scan, equal allele frequencies were assumed while for fine mapping, allele frequencies were estimated from 100 unrelated and unaffected individuals from the Punjab province of Pakistan.

### Mutation screening

Individual exons were amplified by PCR using primer pairs designed by using the primer3 program (primer sequences and annealing temperatures are available upon request). Amplifications were performed in 25 μl reactions containing 50 ng of genomic DNA, 2.5 μl 10X GeneAmp PCR Buffer II, 8 pmoles of each primer, 2.5 mM dNTP, 2.5 mM MgCl_2_, and 0.2 U Taq DNA polymerase. Amplification was performed in a GeneAmp PCR System 9700 (Applied Biosystems). PCR amplification consisted of a denaturation step at 96 °C for 5 min followed by 40 cycles, each cycle starting at 96 °C for 45 s followed by 57 °C for 45 s and 72 °C for 1 min. PCR products were analyzed on 2% agarose gel and purified by ethanol precipitation. The PCR primers for each exon were used for bidirectional sequencing using Big Dye Terminator Ready reaction mix (Applied Biosystems) according to manufacturer instructions. Sequencing products were precipitated and resuspended in 10 μl of formamide and denatured at 95 °C for 5 min. Sequencing was performed on an ABI PRISM 3100 Automated sequencer (Applied Biosystems). Sequencing results were assembled by the ABI PRISM sequencing analysis software version 3.7 and analyzed using Chromas software (version 1.45).

## Results

The two families reported here, PKGL005 and PKGL025, are from the Punjab province of Pakistan. Ophthalmic examinations and medical history for both families concluded that a total of 11 affected individuals in both families have primary congenital glaucoma (PCG). The symptoms of PCG in affected individuals of PKGL005 appeared in the first three years of life. Visual acuity was confined to light perception and/or counting fingers. The cup to disc ratios of affected individuals 11 and 41 were 0.8 (OD) and 1.0/0.3 (OD/OS), and the recorded IOPs for individuals 11 and 41 were 32/20 mm Hg (OD/OS) and 38/30 mm Hg (OD/OS), respectively (Table 1). On the other hand, symptoms of PCG in PKGL025 were either present at birth or appeared in the first six weeks of life. Visual acuity was reduced to counting figures and/or light perception with bilateral buphthalmos eyes. The IOPs for affected individuals in PKGL025 were either above the normal range or was controlled by medical treatment (Table 1).

**Table 1:** Clinical features of affected individuals of families PKGL005 and PKGL025.

**Family number**	**Individual ID**	**Gender**	**Age of onset**	**Age at time of study**	**Maximum IOP (OD/OS)**	**C/D ratio (OD/OS)**	**Visual acuity (OD/OS)**	**Other changes**
PKGL005	11	M	3 years	8 years	32/20*	0.8/NV	CF/CF	Megalocornea
PKGL005	41	M	3 years	6 years	38/30	1.0/0.3	CF/CF	Megalocornea
PKGL025	15	F	By birth	4 years	20*/24*	0.3/0.3	CF/CF	Buphthalmos
PKGL025	28	M	By birth	8 months	25/26	NA	CF/CF	Buphthalmos
PKGL025	25	F	By birth	5 years	12*/16*	0.9/0.9	CF/CF	Megalocornea, cornea haze
PKGL025	24	F	By birth	15 years	NA/38	NV/1.0	NPL/NPL	Buphthalmos

An asterisk indicates that IOP is controlled by medical or surgical treatment. IOP, intraocular pressure; OD, right eye; OS, left eye; PL, perception of light; NPL, no perception of light; HM, hand motion; NA, not available; NV, no view because of eyeball atrophy or corneal opacity; CF, counting fingers.

Initially, all reported loci for PCG were excluded for linkage using closely spaced microsatellite markers (data not shown). A genome wide scan was completed with the ABI MD10 panel, which consisted of 382 polymorphic microsatellite markers and spaced at an average of 10 cM across the whole genome. During the genome-wide scan, LOD scores above 1.5 were obtained for markers D6S308, D10S59, D10S1652, D11S1314, D14S74, D14S68, D16S404, and D18S53 in PKGL005 and for markers D2S112, D3S1279, D9S1776, D14S74, and D21S263 in PKGL025. Of these markers, D6S308, D10S59, D10S1652, D11S1314, D16S404, and D18S53 have closely flanking markers yielding large negative LOD scores in PKGL005. Similarly, in PKGL025, D2S112, D3S1279, D9S1776, and D21S263 have closely flanking markers yielding large negative LOD scores. Linkage to markers other than chromosome 14q markers that showed LOD scores greater than 1.5 during the genome-wide scan was further excluded by haplotype analysis of closely flanking markers.

Two point linkage analysis provided the first evidence of linkage to markers at 14q24.2–24.3 with maximum LOD scores of 5.88 and 6.19 with markers D14S61 and D14S43 at θ=0 for families PKGL005 and PKGL025, respectively. Additional STR markers selected from the NCBI and Marshfield databases were genotyped to define the linkage interval in these families. Two point LOD scores of 4.96, 5.60, 4.01, 4.84, 4.76, 5.88, 3.50, and 3.69 with D14S77, D14S43, D14S284, D14S1036, D14S85, D14S61, D14S59, and D14S1008 at θ=0 were obtained for PKGL005 (Table 2). Similarly, two point LOD scores 4.66, 6.19, 4.44, 5.28, 3.19, and 5.38 with D14S77, D14S43, D14S284, D14S1036, D14S85, and D14S74 at θ=0 were obtained for PKGL025 (Table 3).

**Table 2:** Two point LOD scores of PKGL005 with chromosome 14q markers.

**Marker**	**cM**	**Mb**	**Two-point LOD score values at recombination fraction (θ=)**									**Zmax**	**θ max**
			**0.00**	**0.01**	**0.03**	**0.05**	**0.07**	**0.09**	**0.10**	**0.20**	**0.30**		
D14S63	69.18	44.71	-5.50	-0.38	0.42	0.70	0.83	0.89	0.90	0.72	0.37	0.90	0.10
D14S258	76.28	50.65	-5.49	-0.38	0.41	0.69	0.83	0.89	0.89	0.72	0.37	0.89	0.09
D14S289	78.20	51.63	0.97	1.89	2.13	2.13	2.06	1.96	1.90	1.14	0.38	2.13	0.03
D14S77	80.82	53.63	4.96	4.84	4.61	4.38	4.15	3.91	3.80	2.62	1.51	4.96	0.00
D14S43	84.16	54.98	5.60	5.49	5.24	4.98	4.73	4.46	4.33	3.02	1.74	5.60	0.00
D14S284	84.69	55.75	4.01	3.89	3.65	3.42	3.18	2.94	2.82	1.72	0.84	4.01	0.00
D14S76	84.69	55.82	1.96	1.88	1.71	1.56	1.39	1.25	1.18	0.59	0.99	1.96	0.00
D14S1036	84.69	55.83	4.84	4.73	4.48	4.23	3.98	3.73	3.61	2.36	1.23	4.84	0.00
D14S85	84.76	–	4.76	4.65	4.40	4.15	3.90	3.65	3.52	2.29	1.17	4.76	0.00
D14S61	86.29	56.37	5.88	5.75	5.50	5.24	4.98	4.71	4.58	3.23	1.90	5.88	0.00
D14S59	87.36	58.11	3.50	3.40	3.20	2.99	2.79	2.59	2.49	1.53	0.75	3.50	0.00
D14S74	87.36	58.70	-0.06	2.95	3.18	3.16	3.06	2.93	2.70	1.96	1.06	3.18	0.03
D14S1008	89.19	59.94	3.69	3.61	3.43	3.24	3.06	2.88	2.79	1.90	1.10	3.69	0.00
D14S606	91.62	–	-0.08	2.19	2.46	2.47	2.41	2.31	2.25	1.54	0.81	2.47	0.05
D14S974	93.76	–	-2.14	0.25	0.60	0.70	0.72	0.71	0.70	0.46	0.21	0.72	0.07

LOD scores were calculated at different θ values for each marker with the FASTLINK version of MLINK from the LINKAGE program package. Maximum LOD scores for each marker were calculated using ILINK.

**Table 3:** Two point LOD scores of PKGL025 with chromosome 14q markers.

**Marker**	**cM**	**Mb**	**Two-point LOD score values at recombination fraction (θ=)**									**Zmax**	**θ max**
			**0.00**	**0.01**	**0.03**	**0.05**	**0.07**	**0.09**	**0.10**	**0.20**	**0.30**		
D14S63	69.18	44.71	-2.50	2.00	2.50	2.75	2.75	2.75	2.75	2.00	1.25	2.75	0.03
D14S258	76.28	50.65	2.50	4.00	4.25	4.25	4.00	4.00	3.75	2.75	1.50	4.25	0.03
D14S289	78.20	51.63	1.56	2.69	2.94	2.94	2.81	2.75	2.69	1.88	1.00	2.94	0.03
D14S77	80.82	53.63	4.66	4.59	4.44	4.28	4.09	3.91	3.81	2.78	1.69	4.66	0.00
D14S43	84.16	54.98	6.19	6.03	5.72	5.44	5.13	4.84	4.69	3.19	1.72	6.19	0.00
D14S284	84.69	55.75	4.44	4.31	4.06	3.81	3.63	3.38	3.25	2.06	1.06	4.44	0.00
D14S76	84.69	55.82	2.32	2.27	2.17	2.07	1.97	1.86	1.81	1.29	0.79	2.32	0.00
D14S1036	84.69	55.83	5.28	5.16	4.91	4.63	4.38	4.13	3.99	2.69	1.44	5.28	0.00
D14S85	84.69	–	3.19	3.13	3.06	2.94	2.81	2.68	2.62	1.81	1.06	3.19	0.00
D14S61	86.29	56.37	-4.34	1.91	2.22	2.25	2.22	2.16	2.09	1.47	0.78	2.25	0.05
D14S59	87.36	58.11	-4.34	5.75	5.75	5.75	5.50	5.50	5.25	4.00	2.50	5.75	0.01
D14S74	87.36	58.70	5.38	5.25	5.00	4.75	4.50	4.25	4.13	2.75	1.50	5.38	0.00
D14S1008	89.19	59.94	-4.34	2.75	3.50	3.75	3.50	3.50	3.50	2.50	1.50	3.75	0.05
D14S606	91.62	–	-4.34	2.16	2.84	3.00	3.03	2.97	2.91	2.09	1.09	3.03	0.07
D14S974	93.76	–	-4.34	1.50	2.19	2.38	2.44	2.44	2.38	1.75	0.94	2.44	0.07

LOD scores were calculated at different θ values for each marker with the FASTLINK version of MLINK from the LINKAGE program package. Maximum LOD scores for each marker were calculated using ILINK.

**Figure 1:**
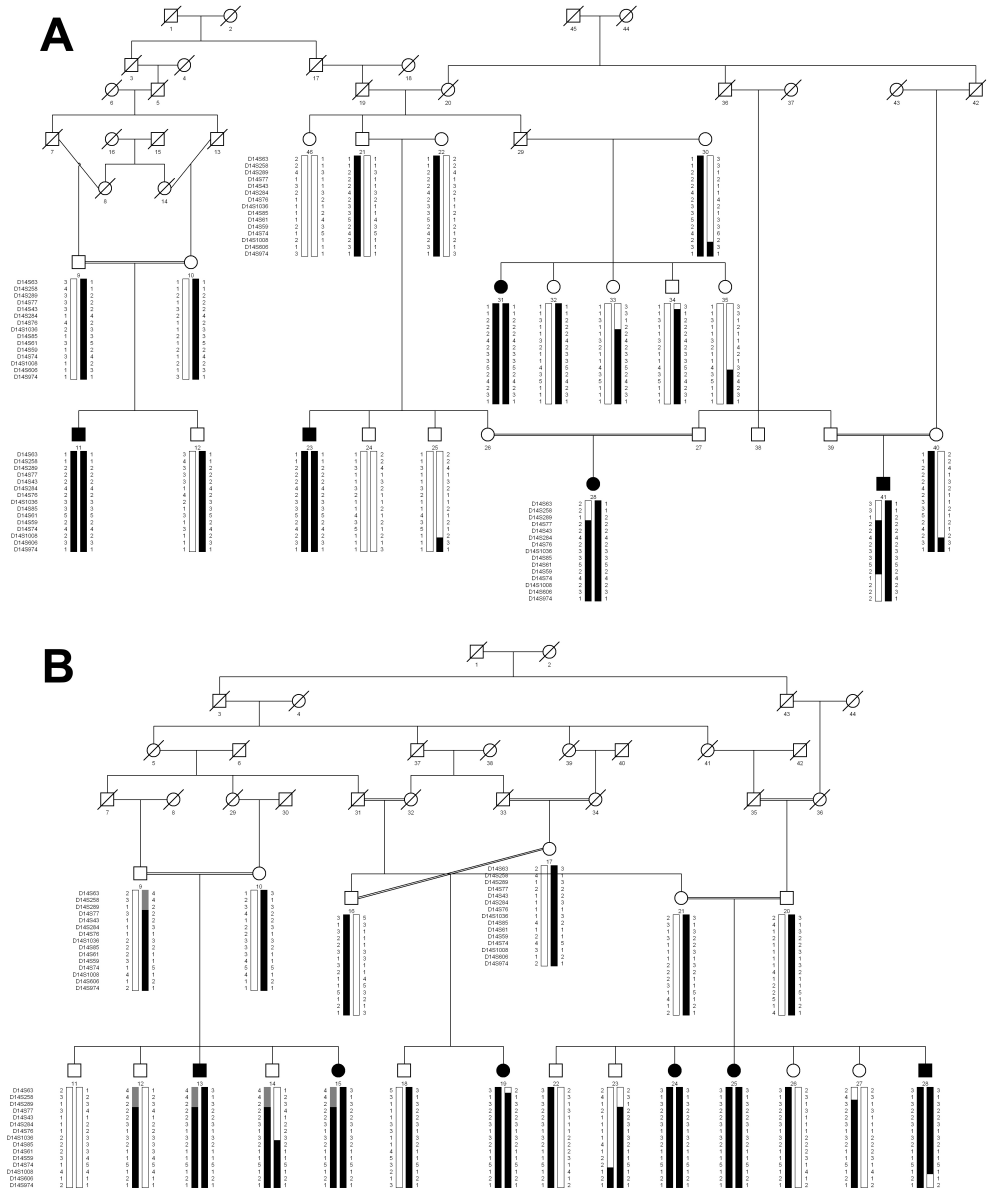
Pedigree of families PKGL005 and PKGL025. Squares denote males, circles indicate females, filled symbols represent affected individuals, double lines between individuals indicate consanguinity, and a diagonal line through a symbol signify that the family member is deceased. The haplotypes of 15 adjacent chromosome 14q14.2–24.3 microsatellite markers for families PKGL005 (**A**) and family PKGL025 (**B**) are shown with alleles forming the risk haplotype shaded black, alleles cosegregating with primary congenital glaucoma (PCG) but not showing homozygosity shaded gray, and alleles not cosegregating with PCG shown in white.

Haplotype analysis supports the results of linkage analysis as shown in Figure 1. There is a proximal recombination in affected individual 19 of PKGL025 at D14S63 and in affected individuals 28 and 41 of PKGL005 at D14S289. Similarly, there is distal recombination in affected individual 28 of PKGL025 at D14S606 and in affected individual 41 of PKGL005 and unaffected individual 23 of PKGL025 at D14S74 as well as in unaffected individual 14 of PKGL025 at D14S85. Taken together, these results suggest the disease locus lies in a 6.56 cM (~4.2 Mb) region flanked by markers D14S289 and D14S85. As marker D14S1036 is uninformative for individual 10 of PKGL025, it is possible that the distal boundary lies proximal to marker D14S1036. Alleles for D14S77, D14S43, D14S284, D14S76, and D14S1036 were homozygous for all affected individuals in families PKGL005 and PKGL025 whereas the normal individuals are either heterozygous carriers of the disease allele or are homozygous for the normal allele.

The critical interval on chromosome 14q24.2–24.3 harbors coenzyme Q6 homolog (*COQ6*), which encodes a flavin-dependent monooxygenase in *Saccharomyces cerevisiae*. This suggests a functional similarity with *CYP1B1*. We investigated the *COQ6* gene to identify the mutation leading to the disease phenotype in these families by sequencing all coding exons, exon-intron boundaries, and the 5'-untranslated region, but we did not find any pathogenic mutations in this gene. Our sequencing results identified previously reported SNPs rs3213692 and rs2074930 in PKGL005 and rs17552038, rs3213692, and rs7141392 in PKGL025.

## Discussion

Here, we report autosomal recessive primary congenital glaucoma (PCG) in two large consanguineous Pakistani families, mapped to chromosome 14q24.2–24.3. Maximum LOD scores of 5.88 and 6.19 with markers D14S61 and D14S43 at θ=0 for families PKGL005 and PKGL025, respectively, the lack of LOD scores above 2.0 for any markers other than chromosome 14q in the entire genome scan, and the disease haplotype segregating with the disease phenotype in both families strongly suggest that the PCG locus maps to chromosome 14q24.2–24.3 in these families. Haplotype analysis of these two families refines the disease interval to a 6.56 cM (~4.2 Mb) region flanked by markers D14S289 and D14S85. Localization of the disease interval to 14q24.2–24.3 in two consanguineous Pakistani families strongly suggests genetic heterogeneity of primary congenital glaucoma.

**Figure 2:**
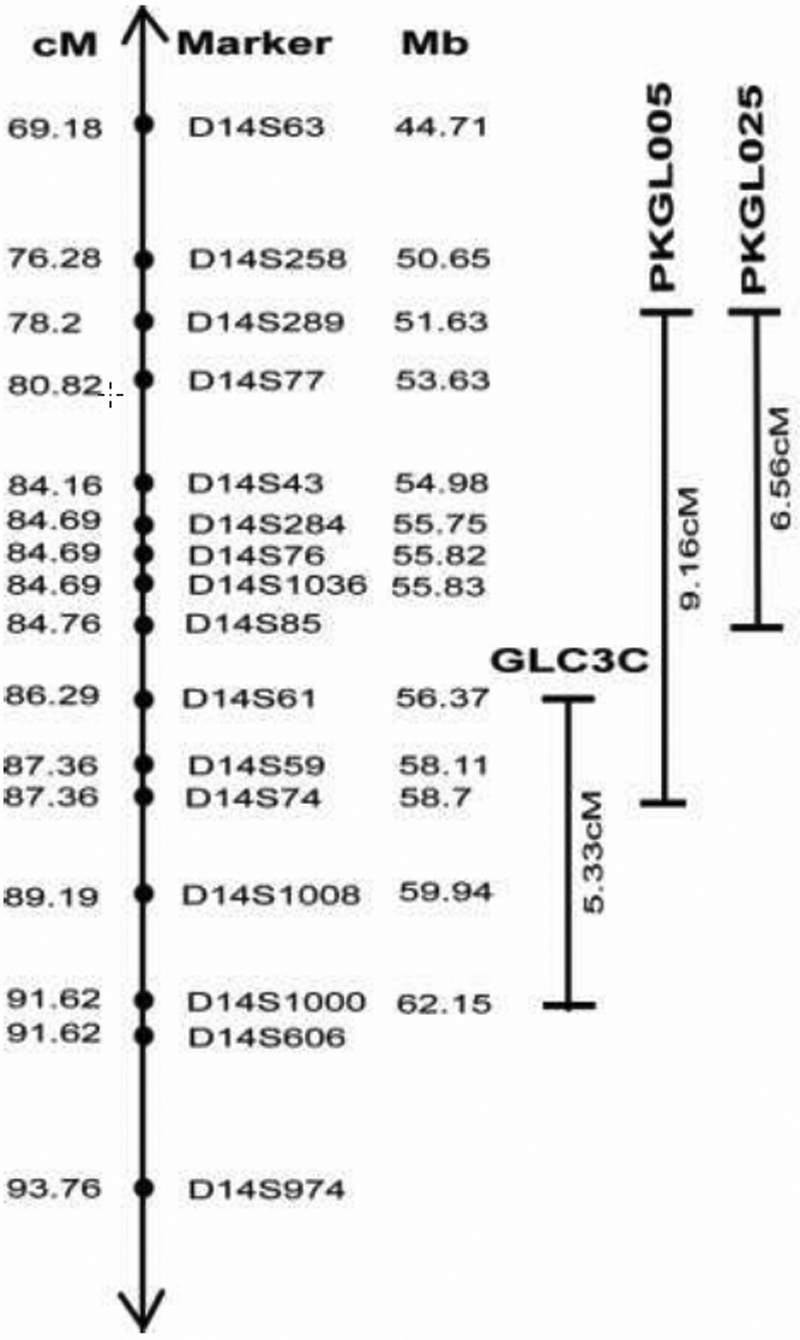
Schematic representation of linkage on chromosome 14q24.2–24.3 in families PKGL005 and PKGL025. Filled circles denote STR markers, and solid vertical lines represent the chromosomal intervals in which markers are homozygous for affected members of each of the two families.

To date, three PCG loci have been mapped to chromosomes 2p21 (*GLC3A*), 1p36 (*GLC3B*), and 14q24.3 (*GLC3C*) whereas mutations associated with PCG have only been reported in the *CYP1B1* gene [13-15,21]. Previously, *GLC3C* was localized to chromosome 14q24.3 flanked by markers D14S61 and D14S1000 as shown in Figure 2 [22]. In PKGL025, individual 14 delineates the distal boundary at marker D14S85, strongly suggesting that the disease locus in PKGL025 does not overlap with *GLC3C*. As both families in this study come from similar geographical and racial backgrounds, haplotype analysis of both families strongly suggests that the region flanked by markers D14S289 and D14S85 harbors the disease causing gene. However, we cannot rule out the possibility that the disease phenotype in these two families is caused by two different mutations, and the pathogenic mutation for PKGL005 may be present in a gene localized in a region overlapping with the *GLC3C* locus.

The critical interval on chromosome 14q24.2–24.3 harbors 97 genes including coenzyme Q6 homolog (*COQ6*), WD repeat domain 21A (*WDR21A*), and ceh-10 homeo domain containing homolog (*CHX10*). COQ6 is a lipid soluble antioxidant and an obligatory component of the respiratory chain and uncoupling proteins [23,24]. *COQ6* in *Saccharomyces cerevisiae* encodes a flavin-dependent monooxygenase, suggesting a functional similarity with *CYP1B1*, a mixed-function monooxygenase that belongs to the cytochrome P450 1B subfamily [25]. We sequenced all the coding exons and exon-intron boundaries as well as the 5’ and 3’ regions of affected individuals of families PKGL005 and PKGL025; however we did not identify any pathogenic mutation.

WDR21A belongs to the WD repeat protein family. Members of this family are involved in a variety of cellular processes including cell cycle progression, signal transduction, apoptosis, and gene regulation. Mutations in *WDR36,* also a member of the WD repeat protein family, have been associated with adult-onset primary open-angle glaucoma (POAG) [26]. In contrast, *CHX10* is homeobox transcription factor gene that is expressed in progenitor cells of the developing neuroretina and in the inner nuclear layer of the mature retina. In humans, *CHX10* mutations are associated with microphthalmia with cataracts and iris abnormalities, isolated microphthalmia with coloboma 3, isolated microphthalmia 2, and isolated microphthalmia with cloudy corneas [27-29]. Similarly, mutations in *CHX10* cause microphthalmia, progressive degeneration of the retina, and an absence of the optic nerve in mice [30]. We are currently sequencing these two candidate genes to identify any pathogenic mutations.

In summary, we have localized autosomal recessive PCG to chromosome 14q24.2–24.3 in two consanguineous Pakistani families. Identification of the PCG causing gene at this locus will help to unveil the underlying molecular complexity of primary congenital glaucoma and will be a valuable addition to the existing repertoire of glaucoma genetics, particularly of PCG. Finally, it will be helpful in screening for carrier status and genetic counseling of PCG families especially in the Pakistani population to prevent severe visual impairment and blindness.
